# Changes in the Profile of Phytochemicals During Processing of Durum Pasta Fortified with Broccoli Leaf Powder and Its Effect on Bioactive Properties

**DOI:** 10.3390/molecules31152672

**Published:** 2026-07-31

**Authors:** Natalia Drabińska-Fois, Mariana Nogueira, Paulina Nowicka, Maja Jeż, Joanna Honke, Cristina Manis, Anna Michalska-Ciechanowska

**Affiliations:** 1 Food Volatilomics and Sensomics Group, Faculty of Food Science and Nutrition, Poznan University of Life Sciences, 60-624 Poznań, Poland; 2 InLife, Institute of Animal Reproduction and Food Research of Polish Academy of Sciences, Trylińskiego 18 Str., 10-683 Olsztyn, Poland; mariana.nogueira95@outlook.pt (M.N.); maja.jez@pum.edu.pl (M.J.); j.honke@pan.olsztyn.pl (J.H.); 3 Department of Fruit, Vegetable and Plant Nutraceutical Technology, Faculty of Biotechnology and Food Science, Wrocław University of Environmental and Life Sciences, Chełmońskiego 37 Str., 51-630 Wrocław, Poland; paulina.nowicka@upwr.edu.pl (P.N.); anna.michalska@upwr.edu.pl (A.M.-C.); 4 PUM Clinical Research Support Center, Unii Lubelskiej 1, 71-252 Szczecin, Poland; 5 Department of Life and Environmental Sciences, University of Cagliari, Cittadella Universitaria di Monserrato, 09042 Monserrato, Italy; cristina.manis@unica.it

**Keywords:** by-product, valorization, broccoli leaves, glucosinolates, durum wheat pasta, functional food, nutritional quality

## Abstract

Broccoli processing generates large quantities of leaves that remain underutilized despite being rich in bioactive compounds. Their valorization as a functional ingredient aligns with sustainable food production and offers an opportunity to enhance the nutritional quality of cereal-based foods. This study evaluated the impact of broccoli leaf powder (BLP) fortification (2.5 and 5%) on the phytochemical composition and functional properties of durum pasta under three drying regimes. BLP introduced glucosinolates, flavonols, carotenoids, and chlorophylls that were absent in the control samples, and their contents increased proportionally with the fortification level. The drying regime modulated compound stability, with low-temperature drying generally favoring retention, whereas cooking caused up to a 50% loss of glucosinolate, depending on the processing conditions. Functional properties were markedly enhanced by BLP addition. The 5% fortification level showed the greatest improvement, resulting in the highest antioxidant activity, antiglycation potential, and angiotensin-converting enzyme (ACE)-inhibitory activity compared to the control pasta. Antioxidant, antiglycation, and ACE-inhibitory activities increased proportionally with the fortification level, and although drying reduced some activities, the fortified pasta consistently outperformed the control. Among the tested formulations, pasta enriched with 5% BLP showed the most favorable overall functional and bioactive compound profiles. Overall, broccoli leaves represent a valuable by-product for developing nutritionally enriched pasta, and the processing conditions play a key role in shaping the stability and bioactivity of the incorporated compounds.

## 1. Introduction

Durum wheat pasta is one of the most widely consumed cereal-based foods globally, valued for its sensory quality, nutritional profile, and technological stability. In recent years, growing consumer interest in functional foods has stimulated efforts to enhance food stuffs with plant-derived ingredients rich in bioactive compounds. Pasta is provides a suitable food matrix for incorporating functional ingredients to obtain products with improved health-beneficial properties without compromising technological quality [1,2,3,4,5]. The obtained products are characterized by improved antioxidant activity and a higher phytochemical content. 

The fruit and vegetable processing industry generates substantial quantities of by-products, many of which remain underutilized despite being rich sources of nutrients and functional phytochemicals [6]. These components, particularly polyphenols, carotenoids, and glucosinolates, are known for their antioxidant and health-promoting properties. Consequently, plant by-products represent a valuable source of potential functional food ingredients [7]. Valorizing these materials as food ingredients offers a dual benefit: increasing the added value of processed foods while contributing to more sustainable production systems. 

Cruciferous crops are among the vegetables with the highest proportion of unharvested or discarded biomass [8]. In the case of broccoli, only 10–15% of the aerial parts are consumed, mainly the florets, whereas the stems and leaves are often treated as waste. Their incorporation into food products could significantly improve waste management and enhance the sustainability of global broccoli production. Although broccoli leaves are rarely accepted for direct consumption, studies consistently demonstrate that they are rich in bioactive compounds, including glucosinolates, polyphenols, and essential vitamins such as E and K [8,9,10,11]. Traditionally used as animal feed or a natural antimicrobial agent, broccoli leaves have recently gained scientific attention due to their high content of phytochemicals and associated biological activities [12,13,14,15]. Importantly, broccoli leaves contain higher concentrations of glucosinolates than the florets, making them a particularly valuable source of health-promoting compounds [11]. Indeed, broccoli leaves exhibit strong antioxidant and anti-inflammatory potential and contain glucosinolate-derived isothiocyanates associated with health-promoting effects [13,16,17]. However, the stability of these compounds is strongly influenced by technological processing. Pasta production involves mixing, extrusion, drying, and cooking, each of which may induce structural and chemical transformations affecting phytochemical retention and biological activity [18]. In particular, drying temperature is known to modulate pigment degradation, polyphenol oxidation, and glucosinolate hydrolysis, while cooking introduces additional thermal and leaching effects that may further alter the functional profile of fortified pasta [19,20]. Although several studies have explored the enrichment of pasta with vegetable powders, data on broccoli leaf powder (BLP) incorporation remain limited, and a comprehensive evaluation of both phytochemical stability and health-relevant bioactivities is lacking. Moreover, most available research focuses primarily on antioxidant capacity [12]. No previous study has simultaneously assessed the behavior of polyphenols, carotenoids, chlorophylls, and glucosinolates in BLP-fortified pasta across different drying regimes. Likewise, antiglycation properties and angiotensin-converting enzyme (ACE) inhibitory activity, two mechanisms relevant to metabolic and cardiovascular health, have not been investigated in this context. 

While the technological and cooking quality characteristics of BLP-fortified pasta were evaluated in our previous study [13,14], the present work focuses on the fate of phytochemicals during processing and on the functional properties of the final product, including antioxidant, antiglycation, and ACE-inhibitory activities. Therefore, this study aimed to investigate the impact of BLP fortification on the phytochemical composition and functional properties of durum pasta, with particular emphasis on the effects of different drying regimes (fresh [F], low-temperature drying at 50 °C for 8 h [L], and high-temperature drying at 80 °C for 3 h [H]) and subsequent cooking. Polyphenols, carotenoids, chlorophylls, and glucosinolates were quantified in the pasta before and after cooking, while antioxidant capacity, antiglycation activity, and ACE-inhibitory potential were evaluated in cooked samples to reflect the actual activity of the bioactive compounds consumed. By integrating phytochemical profiling with multiple bioactivity assays, this study provides novel insights into the valorization of broccoli by-products and elucidates how processing conditions influence the nutritional and nutraceutical value of BLP-enriched pasta.

## 2. Results and Discussion

### 2.1. Phytochemical Composition of Pasta During Processing

#### 2.1.1. Glucosinolate Composition of Pasta During Processing

The technological and cooking quality characteristics of BLP-fortified pasta, including texture, elasticity, swelling behavior, and cooking performance, were comprehensively evaluated in our previous study [14]. Therefore, the present work focuses on the changes in phytochemical composition and functional properties of pasta during processing and cooking.

The glucosinolate contents of BLP and the pasta products are presented in Table 1. In BLP, five glucosinolates were identified, with indole glucosinolates being the predominant group. Glucosinolates characteristic of broccoli [21], such as glucoraphanin, were also detected. The same glucosinolates were identified in pasta products enriched with BLP. Neoglucobrassicin was the dominant glucosinolate in fortified pasta, similarly to BLP. The total content of individual glucosinolates increased proportionally with the level of BLP addition. 

The drying conditions affected the glucosinolate content in different ways depending on the level of BLP addition. In B2.5 pasta, the L drying regime resulted in the complete degradation of progoitrin. By contrast, glucoraphanin and indole glucosinolates remained at levels comparable to those observed in fresh samples. The H drying regime resulted in a higher progoitrin content in B2.5 pasta than in fresh pasta. This observation may be associated with improved extractability or other matrix-related effects induced by processing rather than actual formation of these compounds. For B5 pasta, the L drying method led to increases in the contents of individual glucosinolates compared to fresh pasta, with the biggest increase in progoitrin levels (3 times). Notably, for B5 samples, all indole glucosinolates were present at higher levels after L drying than in fresh pasta and after H drying.

Cooking resulted in significant losses of all glucosinolates, with no major differences observed between individual compounds or their groups. In fresh pasta, glucosinolate losses ranged from 8 up to 54%. In dried pasta samples (both L and H regimes), cooking caused similar losses. The observed losses of glucosinolates are in accordance with previous studies reporting that thermal processing causes the degradation of glucosinolates [21,22,23,24,25]. Some of the observed increases during processing can be explained by the improved extractability of these compounds, not *de novo* synthesis, as was also observed in previous studies [24,25].

#### 2.1.2. Polyphenolic Composition of Pasta During Processing

The phenolic compound contents of BLP and the pasta samples are presented in Table 2. BLP was characterized by high contents of flavonols and phenolic acids, both reaching nearly 2000 mg/kg DW, whereas flavan-3-ols were present at much lower levels.

Similar to glucosinolates, the phenolic compounds content of pasta increased with the level of BLP addition. The control pasta (without BLP) contained no flavonols, only small amounts of phenolic acids and relatively high levels of flavan-3-ols.

Drying influenced the phenolic compound content in a compound-dependent manner. For flavonols, in B2.5 pasta, both drying methods resulted in their higher contents compared to fresh pasta. In B5 samples, a slight increase was observed after L drying, whereas H drying resulted in levels comparable to those in fresh pasta. In the case of phenolic acids, both B2.5 and B5 samples showed decreases in their contents with increasing drying temperature, indicating their sensitivity to thermal processing. Flavan-3-ols exhibited a different pattern. The addition of BLP resulted in slight but noticeable increases in their contents, which tended to rise with higher levels of enrichment. In B2.5 pasta, levels of flavan-3-ols were relatively stable, with only minor fluctuations observed across drying treatments. However, in B5 samples, an increase in drying temperature led to a significant reduction in flavan-3-ol content.

Cooking resulted in losses of most phenolic compounds, ranging from 4 to 42% for phenolic acids, from 2 to 40% for flavan-3-ols, and generally substantial decrease in flavonol content. Flavonol levels lowered markedly in all samples, with the exception of B5 pasta after L drying, where an increase of 26% was observed after cooking. The losses of phenolic acids varied depending on the sample: 25% in fresh B2.5 pasta, 42% in B2.5-L, and 36% in B2.5-H. For B5 samples, the decreases were smaller, amounting to 4% in fresh pasta, 27% for L drying, and 26% for H drying. The behavior of flavan-3-ols during cooking was less uniform. In control and B5 pasta dried under the high temperature conditions, their contents increased by 10 and 33%, respectively. In the remaining samples, decreases were observed, ranging from 2% (B5-L) to as much as 40% (B2.5-F). This decrease in the levels of phenolic compounds, although expressed as the total phenolic content (TPC) and total flavonoid content, was reported in previous studies [5,26]. The observed losses are mainly attributed to the thermal degradation of heat-sensitive phenolic constituents and their leaching into the cooking water due to the water-soluble nature of many phenolic compounds. In addition, thermal processing may promote oxidation reactions and interactions between phenolics and macromolecules present in the pasta matrix, reducing their extractability and resulting in lower measured concentrations. The authors underline that these losses are highly dependent on the matrix; therefore, the optimization of drying conditions seems to be crucial for maintaining bioactive compounds.

#### 2.1.3. Chlorophyll Composition of Pasta During Processing

The chlorophyll pigment contents are presented in Table 3. No chlorophylls were detected in the control pasta. In BLP, chlorophyll A and pheophytin A were identified, confirming the presence of green plant pigments and their derivatives.

In pasta products, the chlorophyll A content increased proportionally with the level of BLP addition. However, drying led to reductions in chlorophyll A content in both B2.5 and B5 samples, regardless of the processing regime, indicating its sensitivity to thermal treatment and degradation. The pheophytin A content ranged from 1.29 to 2.34 mg/kg. Although higher levels were observed in B5 compared to B2.5 samples, the increase (approximately 9%) was not proportional to the increase in BLP addition. Furthermore, at both enrichment levels, increasing the drying temperature resulted in a decrease in pheophytin A content.

Cooking further affected chlorophyll stability, resulting in chlorophyll A decrease of approximately 16–50% in most samples, whereas changes in pheophytin A levels ranged from slight increases (16–20%) in fresh pasta to losses of up to 30% in dried samples. In B2.5 pasta after H drying, chlorophyll A was no longer detectable after cooking. In the remaining samples, its content decreased to varying extents. In B2.5 pasta, the loss reached approximately 50%, whereas in B5 samples, reductions were smaller: 23% in fresh pasta and 16% in pasta after L drying. Interestingly, in B5-H, the chlorophyll A content remained at a level comparable to that before cooking, which may again be associated with matrix-related changes affecting extractability. 

By contrast, pheophytin A showed a different behavior during cooking. In fresh pasta, its content increased by 2% in B2.5 and 16% in B5 samples, suggesting the conversion of chlorophylls into pheophytins under thermal conditions. Similarly, an increase in its content was observed in previous studies with various vegetables [27]. In dried pasta, moderate losses of pheophytin A were observed, generally ranging from 1 to 30%. The exception was B2.5-H pasta, in which pheophytin A was not detected after cooking.

#### 2.1.4. Carotenoid Composition of Pasta During Processing

No carotenoids were detected in the control pasta, confirming that durum semolina does not contribute to measurable amounts of these pigments. By contrast, BLP contained 11 carotenoids, including violaxanthin, astaxanthin, capsanthin, lutein, cryptoxanthin derivative, α-cryptoxanthin, β-cryptoxanthin, δ-carotene, γ-carotene, ζ-carotene, and β-carotene (Table 4). Among these, β-carotene, lutein, and δ-carotene exhibited the highest concentrations, reflecting the characteristic carotenoid profile of broccoli leaves.

B2.5 pasta contained eight carotenoids, namely lutein, cryptoxanthin derivative, α-cryptoxanthin, β-cryptoxanthin, δ-carotene, γ-carotene, ζ-carotene, and β-carotene. The dominant compounds were lutein and β-carotene, whereas the remaining carotenoids were present at trace levels. Increasing the fortification level to 5% BLP (B5) resulted in the detection of all 11 carotenoids, including violaxanthin, astaxanthin, and capsanthin, which were absent in B2.5. However, an increase in BLP addition did not uniformly enhance carotenoid concentrations. Surprisingly, lutein, γ-carotene, ζ-carotene, and β-carotene levels were higher in B2.5 than in B5, suggesting matrix-dependent interactions or differential stability at higher incorporation levels.

The drying conditions exerted a distinct influence on carotenoid retention, and the magnitude and direction of these changes depended both on the fortification level and on the specific compound. In B2.5 pasta, the low-temperature drying regime resulted in higher concentrations of α-cryptoxanthin and γ-carotene compared to fresh samples, indicating that mild dehydration may facilitate pigment release from the matrix or limit oxidative degradation. By contrast, the high-temperature drying regime caused only minor losses of carotenoids or slight increases in their levels in B2.5 relative to fresh pasta, suggesting that the intensified thermal load did not substantially compromise carotenoid stability at this fortification level. A more pronounced effect was observed in B5 pasta. L drying enhanced the levels of astaxanthin, capsanthin, lutein, and β-cryptoxanthin, demonstrating that gentle dehydration favored the retention of both xanthophylls and carotenes. H drying, however, led to clear increases in the levels of several carotenoids, including lutein, the cryptoxanthin derivative, γ-carotene, ζ-carotene, and β-carotene, when compared to fresh pasta. This pattern may indicate the occurrence of matrix-related effects influencing compound recovery during analysis or possible transformations of carotenoid precursors during processing. However, these mechanisms were not directly examined in the present study. The contrasting responses between B2.5 and B5 indicate that the interaction between drying temperature regimes and carotenoid stability is strongly matrix-dependent, and that the structural characteristics of pasta enriched with higher levels of BLP may modulate pigment release and transformation during dehydration.

Cooking influenced the carotenoid profile of the fortified pasta in a compound-specific manner, and the direction of these changes differed between the two fortification levels. In both B2.5 and B5 pasta, cooking generally led to moderate losses of most xanthophylls and minor carotenoids, which is consistent with the known thermal sensitivity of these pigments and the possibility of leaching into the cooking water. Despite these expected reductions, the behavior of carotenes was markedly different. In cooked fresh pasta, the concentrations of δ-carotene, γ-carotene, ζ-carotene, and β-carotene increased relative to uncooked samples. These increases suggest that the cooking process may have enhanced the extractability of these compounds by softening the pasta matrix or disrupting pigment–protein interactions, thereby facilitating their release during analytical extraction. It is also plausible that thermal treatment promoted partial conversion of carotenoid precursors into more stable carotene forms, contributing to the observed rise in measurable content. The conversion of carotenoids during thermal treatment has been previously reported [28]. The overall pattern indicates that, while cooking inevitably reduces the levels of several oxygenated carotenoids, the carotenes present in BLP-fortified pasta exhibit greater thermal resilience and may even benefit from the structural modifications induced by boiling. These findings highlight the importance of considering both degradation and transformation phenomena when evaluating carotenoid stability in thermally processed cereal matrices enriched with vegetable by-products.

A comparison of the different phytochemical groups revealed distinct responses to cooking. Glucosinolates were the most susceptible compounds, with losses reaching up to 50%. Levels of phenolic compounds also decreased after cooking, although the magnitude of the loss depended on the specific subclass and processing conditions. Chlorophyll pigments showed moderate thermal instability, with chlorophyll A being more susceptible to degradation than its derivative pheophytin A, which in some cases showed increased levels as a result of thermal conversion. By contrast, carotenoids exhibited the highest apparent stability during cooking, and several carotenes showed increased extractable concentrations after thermal treatment. The observed increases in the measured concentrations of selected phytochemicals are unlikely to result from de novo synthesis and may instead reflect changes in analytical recovery, extractability, or compound transformations occurring during processing. However, additional studies would be required to verify the contributions of these mechanisms. Additionally, thermal treatment may promote the conversion of certain phytochemicals into more readily detectable derivatives, as observed for pheophytin formation from chlorophylls. Overall, the stability of phytochemicals during processing appears to depend not only on their thermal sensitivity but also on matrix-related effects influencing their release and analytical recovery.

### 2.2. Functional Properties of Cooked Pasta

Functional properties were evaluated exclusively in cooked pasta to reflect the actual intake of bioactive compounds by consumers. BLP exhibited pronounced antioxidant capacity, as confirmed by both the Trolox Equivalent Antioxidant Capacity (TEAC) by 2,2-azinobis-(3-ethylbenzothiazoline-6-sulphonic acid) diammonium salt (ABTS) as well as ferric reducing antioxidant potential (FRAP) assays, and showed a high TPC values. The corresponding data are presented in Table 5. By contrast, the control pasta displayed slight antioxidant capacity, which further decreased with increasing drying temperature, indicating that the thermal treatment applied during dehydration negatively affected the residual antioxidant potential of the semolina matrix.

Fortification with BLP markedly enhanced the antioxidant properties of the pasta compared to control samples, and the magnitude of this improvement was proportional to the level of addition, which is in agreement with previous studies [3,5,12]. Both TEAC ABTS and TPC values increased with higher BLP incorporation, demonstrating that broccoli leaf constituents effectively enriched the pasta with phenolic compounds and other antioxidant-active molecules. However, the influence of the drying temperature regimes varied depending on the assay and fortification level. For TEAC ABTS and TPC, H drying consistently resulted in lower values, suggesting that the phenolic compounds and ABTS-reactive antioxidants were particularly sensitive to thermal degradation. The FRAP assay revealed a more nuanced pattern. In B2.5 pasta, the low- and high-temperature drying regimes produced comparable FRAP values, indicating that the ferric-reducing capacity of the matrix was relatively stable across the applied thermal conditions. In B5 pasta, FRAP values remained stable after L drying but decreased following H drying, suggesting that at higher fortification levels, the intensified thermal treatment may have exceeded the stability threshold of certain reducing compounds. Overall, these results demonstrate that BLP fortification substantially improves the antioxidant potential of cooked pasta, while the drying regime modulates the extent of this enhancement in compound- and assay-specific manners.

Antiglycation activity was evaluated in cooked pasta using two complementary in vitro models, BSA-glucose (BSA-GLU) and BSA-methylglyoxal (BSA-MGO), and the results are expressed as percentage of inhibition (Table 6). BLP exhibited exceptionally strong antiglycation potential, exceeding 90% inhibition in both models, which confirms the high reactivity of its phenolic constituents and glucosinolate-derived metabolites toward glycation intermediates. By contrast, the control pasta showed only minimal antiglycation activity in the BSA-glucose model and no measurable inhibition in the BSA-MGO model, indicating that semolina alone provides negligible protection against protein glycation.

Relative to the control pasta, fortification with BLP markedly enhanced the antiglycation properties of the pasta, and the magnitude of this improvement depended on both the fortification level and the drying conditions. In the BSA-glucose model, B2.5 pasta exhibited decreasing inhibition with increasing drying temperature, suggesting that the compounds responsible for glucose-derived glycation inhibition were sensitive to thermal degradation. In B5 pasta, drying resulted in a different pattern: both the low- and high-temperature drying regimes slightly increased antiglycation activity compared to the fresh pasta, indicating that at higher incorporation levels, the matrix may better protect or release antiglycation-active compounds during dehydration.

In this study, the BSA-GLU and BSA-MGO models were employed to evaluate the anti-advanced glycation end products (AGE) activity of the analyzed extracts obtained from BLP-fortified pasta (Table 6). These in vitro models are widely recognized as reliable tools for assessing the inhibitory effects of plant foods on the non-enzymatic formation of AGEs [29].

The BSA-MGO model revealed additional differences between fortification levels. In B2.5 pasta, L drying reduced antiglycation activity, whereas H drying resulted in inhibition comparable to that of the fresh sample. This suggests that MGO-reactive compounds in moderately fortified pasta may be more susceptible to mild thermal treatment than to intense dehydration. In B5 pasta, antiglycation activity increased progressively with increase in drying temperature, indicating that higher thermal load may enhance the extractability or stability of compounds capable of trapping MGO or inhibiting AGE formation. Together, these findings demonstrate that the antiglycation response of fortified pasta is strongly model-dependent and influenced by both the concentration of broccoli leaf constituents and the applied processing conditions. The analyzed phytochemicals, like polyphenols, have been reported to exert strong antiglycation properties [30]. 

ACE-inhibitory activity, expressed as IC_50_ values, further confirmed the functional potential of BLP fortification (Table 6). The control pasta required the highest concentration to reach IC_50_, reflecting its weak inhibitory effect. By contrast, BLP exhibited a very low IC_50_ value, indicating potent ACE inhibition. Incorporation of BLP substantially reduced the concentration required to achieve IC_50_ in fresh pasta, demonstrating a clear dose-dependent improvement in ACE-inhibitory potential. Drying, however, consistently increased the IC_50_ values at both fortification levels, indicating a reduction in inhibitory activity as the thermal load intensified. This pattern suggests that ACE-inhibitory compounds present in BLP are sensitive to dehydration, and that higher drying temperatures diminish their bioactivity despite the overall functional enhancement provided by BLP fortification.

ACE plays a central role in the local renin–angiotensin system by cleaving the C-terminal His–Leu dipeptide from angiotensin I, thereby generating angiotensin II and promoting an increase in blood pressure [31]. Consequently, ACE inhibition represents a key therapeutic approach for managing hypertension, a widespread condition associated with severe cardiovascular, neurological, and renal complications. Synthetic ACE inhibitors, including lisinopril, enalapril, and lenopril, are commonly prescribed for hypertension, but their prolonged use may lead to adverse reactions such as hypersensitivity or kidney dysfunction [32]. Naturally derived ACE inhibitors offer a promising alternative, potentially reducing these risks and providing safer treatment options. The pasta fortified with BLP showed an enhanced in vitro ACE-inhibitory activity compared to control pasta.

## 3. Materials and Methods

### 3.1. Pasta Preparation

The preparation of BLP was previously described by Drabińska et al. [12]. The leaves of broccoli (*Brassica oleracea* L. var. *italica* cv. Sebastian) were donated by the fruit and vegetables warehouse GEMIX (Olsztyn, Poland). Leaves were washed in tap water to remove soil residues and blanched in boiling water for 1 min to inactivate enzymes. The material was freeze-dried and ground into a fine powder with a particle size of ≤0.60 mm. BLP was stored in darkness in a refrigerator in a tightly closed container until analysis.

The pasta preparation procedure, detailed formulations, and technological characteristics of the pasta products can be found elsewhere [13,14]. Briefly, the dough was produced using durum semolina, water, olive oil, and salt, all sourced from local retailers. Broccoli leaf powder (BLP) was incorporated into the standard control formulation at three levels: 0% (C), 2.5% (B2.5), and 5% (B5). The ingredients were blended for 5 min in an electric pasta maker (Ariete Pastamatic 1581, De’Longhi, Florence, Italy), after which the dough was extruded through a penne-shaped die using the same device. 

For each formulation, three pasta variants were obtained, differing in drying conditions: (F) fresh, non-dried pasta; (L) pasta dried at 50 °C for 8 h; and (H) pasta dried at 80 °C for 3 h. All samples were cooked to their previously determined optimal cooking time (OCT) [14]. Both uncooked and cooked pasta were subsequently freeze-dried, milled to a fine powder with a particle size of ≤0.60 mm, and stored in airtight containers under refrigeration until further analyses.

### 3.2. The Analysis of Glucosinolates

The glucosinolate content was analyzed as described previously [33], following the method of the Official Journal of the European Communities [34]. Individual glucosinolates were identified and quantified in response to the commercial standards purchased from PhytoPlan® (Heidelberg, Germany).

### 3.3. Polyphenol Compounds Analysis

The polyphenol content was analyzed as described previously [35]. 

Polyphenols were quantified using an ACQUITY Ultra-Performance Liquid Chromatography (UPLC) system (Waters Corporation, Milford, MA, USA) equipped with a binary solvent manager and a photodiode array (PDA) detector. Separation was performed on an ACQUITY UPLC BEH C18 column (2.1 × 100 mm, 1.7 μm; Waters Corporation, Milford, MA, USA) maintained at 30 °C. The injection volume was 5 μL, the flow rate was 0.42 mL/min, and the chromatographic separation was completed within 30 min using 2.0% formic acid in water (solvent A) and acetonitrile (solvent B) as the mobile phases. Phenolic acids, flavonols, and flavan-3-ols were monitored at 320, 360, and 280 nm, respectively.

Chromatographic data were processed using Empower 3 software (Waters Corporation, Milford, MA, USA). Quantification was performed using external calibration curves prepared with authentic commercial standards of the major phenolic compounds representative of broccoli leaves, including *p*-coumaric acid and ferulic acid (phenolic acids); isorhamnetin, quercetin, and kaempferol-3-*O*-rutinoside (flavonols); as well as (+)-catechin and (–)-epicatechin (flavan-3-ols), where applicable. 

### 3.4. Carotenoid and Chlorophyll Analysis

The contents of carotenoids and chlorophylls were analyzed following the method described elsewhere [35,36]. Chromatographic analysis was performed using an ACQUITY Ultra-Performance Liquid Chromatography (UPLC) system (Waters Corporation, Milford, MA, USA) equipped with a photodiode array (PDA) detector. Separation was carried out on an ACQUITY UPLC BEH RP C18 column (2.1 × 100 mm, 1.7 μm; Waters Corporation, Milford, MA, USA) fitted with a C18 guard column and maintained at 32 °C. The injection volume was 10 μL. The mobile phase consisted of 0.1% formic acid in water (solvent A) and acetonitrile (7:3, *v*/*v*) (solvent B).

Chromatographic data were processed using Empower 3 software (Waters Corporation, Milford, MA, USA). Individual carotenoids and chlorophyll derivatives were identified by comparing their retention times and UV–Vis absorption spectra with those of authentic commercial reference standards analyzed under identical chromatographic conditions. Quantification was performed using external calibration curves prepared from the corresponding authentic standards. Chlorophylls and their derivatives were quantified at 430 nm, whereas carotenoids were quantified at 450 nm.

### 3.5. Antioxidant Activity

A total of 150 mg of the freeze-dried sample was extracted with 1 mL of methanol–water solution (80:20; *v*/*v*). Ultrasonic vibration (30 s) and vortexing (30 s) were repeated three times, and the samples were centrifuged for 10 min at 13,000 rpm at 4 °C. The above step was repeated five times, and the supernatants were collected in a 5 mL measuring flask. Methanol extracts were prepared in triplicate and were used for all antioxidant capacity assays. Total phenolic content (TPC) was determined as described previously [33]. The FRAP assay was performed according to the method proposed by Horszwald and Andlauer [37] and described previously [33]. 6-Hydroxy-2,5,7,8-tetramethylchroman-2-carboxylic acid (Trolox) was used for calibration, and the results were expressed in µmol TE (Trolox equivalents)/g DM. The TEAC assay was performed based on the method described by Horszwald and Andlauer [37] using a ABTS solution. Trolox was used for standard calibration, and the results were expressed in µmol TE/g DM.

### 3.6. Anti-Glycation Properties

The inhibitory activity against the formation of AGE products was evaluated in vitro as the ability to inhibit the adduct formation between bovine serum albumin and glucose (BSA-glucose) and bovine serum albumin and methylglyoxal (BSA-MGO) according to the method described by Błaszczak et al. [38]. About 300 mg of powdered pasta sample was extracted with 1 mL of 80% MeOH, shaken for 2 h at 25 °C, and centrifuged (16,000× *g* for 10 min), and the supernatant was collected and dried under nitrogen. The samples were dissolved in solvents for the anti-AGE formation assay. The assays for BSA-glucose and BSA-MGO were measured using a microplate reader (Infinite M1000 PRO, Tecan Group AG, Männedorf, Switzerland) in fluorescence mode set at excitation/emission wavelengths of 360/420 nm and 340/420 nm, respectively.

### 3.7. Angiotensin-I Converting Enzyme (ACE) Inhibitory Activity Assay

The inhibitory effects of pasta extracts on ACE activity were analyzed using an assay described by Sentandreu and Toldrá [39]. Briefly, 50 μL of ACE solution (7.5 mg/mL in 150 mM Tris-base buffer, pH 8,3) was added to each microplate well, the volume was adjusted to 100 μL by adding deionized water (control), and the methanolic pasta extracts or captopril (0.5–3 μg/mL, as a positive control) were added. The enzymatic reaction was initiated by adding 200 μL of 0.45 mM Abz-Gly-Phe(NO_2_)-Pro dissolved in 150 mM tris-base buffer (8.3 pH) containing 1.125 M NaCl. The mixture was immediately stirred and incubated at 37 °C. Fluorescence measurements were carried out using a microplate reader upon achievement of the incubation temperature (0 min) and after 30 min at λ = 355 nm (excitation wavelength) and λ = 405 nm (emission wavelength).

### 3.8. Statistical Analysis

A limitation of the present study is that each pasta formulation was produced as a single batch; therefore, the reported variability reflects analytical replication rather than batch-to-batch variation. All analytical measurements were performed in triplicate on homogenized samples obtained from a single production batch of each pasta variant and are expressed as the mean ± standard deviation. The results were analyzed using a one-way analysis of variance (ANOVA). The significance of differences between the samples was determined by Fisher’s LSD test at *p*-value < 0.05. All statistical analyses were performed using STATISTICA version 14.3.0.6 (Cloud Software Group, Inc, Fort Lauderdale, FL, USA) software.

## 4. Conclusions

This study demonstrates that BLP is a promising ingredient for durum pasta fortification and an effective approach to the valorization of broccoli processing by-products. BLP-enriched pasta contained polyphenols, carotenoids, chlorophylls, and glucosinolates, resulting in improved antioxidant, antiglycation, and ACE-inhibitory properties.

Processing conditions significantly affected phytochemical retention and in vitro biological activity, highlighting the importance of adjusting drying and cooking parameters. Nevertheless, BLP-fortified pasta consistently exhibited superior functional properties compared to the control samples.

Overall, broccoli leaves represent a sustainable and nutritionally valuable raw material for the development of cereal-based foods with enhanced phytochemical content and improved in vitro biological activity. Further studies are needed to better characterize the transformation products formed during processing and their contribution to the biological activity of the final product.

## Figures and Tables

**Table 1:** The glucosinolate contents of broccoli leaf powder (BLP) and pasta products expressed as µmol/g DW.

	Progoitrin	Glucoraphanin	Glucobrassicin	4-Methoxy- Glucobrassicin	Neoglucobrassicin
BLP	0.17 ± 0.04	0.49 ± 0.02	0.86 ± 0.03	0.60 ± 0.02	1.02 ± 0.06
Uncooked pasta					
C-F	-	-	-	-	-
C-L	-	-	-	-	-
C-H	-	-	-	-	-
B2.5-F	0.011 ± 0.003 ^b^	0.013 ± 0.001 ^d^	0.023 ± 0.000 ^d,e^	0.015 ± 0.001 ^e^	0.026 ± 0.000 ^d^
B2.5-L	-	0.005 ± 0.000 ^f^	0.018 ± 0.001 ^f^	0.012 ± 0.001 ^f^	0.026 ± 0.000 ^d^
B2.5-H	0.014 ± 0.006 ^a,b^	0.012 ± 0.002 ^d^	0.020 ± 0.002 ^e,f^	0.013 ± 0.001 ^e,f^	0.024 ± 0.002 ^d^
B5-F	0.005 ± 0.001 ^c^	0.021 ± 0.003 ^a^	0.038 ± 0.006 ^a,b^	0.025 ± 0.004 ^a,b^	0.044 ± 0.008 ^a^
B5-L	0.006 ± 0.001 ^c^	0.021 ± 0.000 ^a^	0.040 ± 0.000 ^a^	0.027 ± 0.001 ^a^	0.044 ± 0.008 ^a^
B5-H	0.015 ± 0.004 ^a,b^	0.020 ± 0.001 ^a,b^	0.036 ± 0.000 ^b^	0.023 ± 0.001 ^b,c^	0.042 ± 0.001 ^a^
Cooked pasta					
C-F	-	-	-	-	-
C-L	-	-	-	-	-
C-H	-	-	-	-	-
B2.5-F	0.005 ± 0.000 ^c^	0.012 ± 0.000 ^d^	0.019 ± 0.001 ^f^	0.012 ± 0.001 ^f^	0.022 ± 0.001 ^d^
B2.5-L	-	0.004 ± 0.001 ^f^	0.019 ± 0.001 ^f^	0.012 ± 0.001 ^f^	0.022 ± 0.001 ^d^
B2.5-H	0.017 ± 0.004 ^a^	0.009 ± 0.000 ^e^	0.013 ± 0.000 ^f^	0.007 ± 0.000 ^g^	0.015 ± 0.001 ^e^
B5-F	0.004 ± 0.001 ^c^	0.021 ± 0.001 ^a^	0.035 ± 0.001 ^b^	0.022 ± 0.000 ^c^	0.040 ± 0.001 ^a,b^
B5-L	0.004 ± 0.001 ^c^	0.018 ± 0.001 ^b,c^	0.030 ± 0.001 ^c^	0.018 ± 0.001 ^d^	0.035 ± 0.002 ^b,c^
B5-H	0.011 ± 0.001 ^b^	0.017 ± 0.001 ^c^	0.026 ± 0.001 ^d^	0.015 ± 0.000 ^e^	0.032 ± 0.000 ^c^

Lowercase letters indicate a significant difference (Fisher’s LSD test at *p*-value < 0.05) between samples. BLP—broccoli leaf powder; C—control; B2.5—pasta fortified with 2.5% BLP; B5—pasta fortified with 5% BLP; F—fresh pasta; L—pasta dried at 50 °C for 8 h; H—pasta dried at 80 °C for 3 h.

**Table 2:** The polyphenolic compound contents of broccoli leaf powder (BLP) and pasta products, expressed as mg/kg DW.

	Flavonols	Phenolic Acids	Flavan-3-ols
BLP	2006.75 ± 120.41	1960.99 ± 117.66	75.61 ± 4.54
Uncooked pasta			
C-F	-	1.48 ± 0.09 ^i^	188.46 ± 11.31 ^e^
C-L	-	1.28 ± 0.08 ^i^	168.22 ± 10.09 ^f^
C-H	-	1.09 ± 0.07 ^i^	101.85 ± 6.11 ^h^
B2.5-F	7.36 ± 0.44 ^f,g^	68.63 ± 4.12 ^b,c^	190.53 ± 11.43 ^d,e^
B2.5-L	9.42 ± 0.57 ^f^	60.31 ± 3.62 ^d^	198.12 ± 11.43 ^c^
B2.5-H	18.03 ± 1.08 ^d^	37.22 ± 2.23 ^g^	196.13 ± 11.77 ^c^
B5-F	34.22 ± 2.09 ^c^	94.10 ± 5.65 ^a^	295.21 ± 17.71 ^a^
B5-L	39.46 ± 2.37 ^b^	73.13 ± 4.39 ^b^	230.60 ± 13.84 ^b^
B5-H	34.86 ± 2.09 ^c^	63.98 ± 3.84 ^c,d^	214.45 ± 12.87 ^b,c^
Cooked pasta			
C-F	-	1.40 ± 0.08 ^i^	207.41 ± 12.44 ^c,d^
C-L	-	1.08 ± 0.06 ^i^	105.15 ± 6.31 ^h^
C-H	-	1.03 ± 0.06 ^i^	135.67 ± 8.14 ^g^
B2.5-F	4.97 ± 0.30 ^g^	51.67 ± 3.10 ^e,f^	114.13 ± 8.51 ^h^
B2.5-L	-	35.11 ± 2.11 ^g^	166.57 ± 9.99 ^f^
B2.5-H	6.98 ± 0.42 ^f,g^	23.79 ± 1.43 ^h^	117.60 ± 7.06 ^g,h^
B5-F	13.16 ± 0.79 ^e^	90.57 ± 5.43 ^a^	230.44 ± 13.83 ^b^
B5-L	49.91 ± 2.99 ^a^	53.22 ± 3.19 ^e^	226.06 ± 13.56 ^b^
B5-H	8.83 ± 0.53 ^f^	47.11 ± 2.83 ^f^	134.28 ± 8.06 ^g^

Lowercase letters indicate a significant difference (Fisher’s LSD test at *p*-value < 0.05) between samples.

**Table 3:** The chlorophyll contents of broccoli leaf powder (BLP) and pasta products, expressed as mg/kg DW.

	Chlorophyll A	Pheophytin A
BLP	71.66 ± 3.58	22.14 ± 1.56
Uncooked pasta		
C-F	-	-
C-L	-	-
C-H	-	-
B2.5-F	2.51 ± 0.13 ^e^	2.14 ± 0.16 ^c,d^
B2.5-L	1.98 ± 0.10 ^f^	1.86 ± 0.13 ^e,f^
B2.5-H	1.20 ± 0.06 ^g^	1.68 ± 0.11 ^f^
B5-F	6.62 ± 0.33 ^a^	2.34 ± 0.17 ^b,c^
B5-L	4.89 ± 0.24 ^b^	1.71 ± 0.11 ^e,f^
B5-H	2.89 ± 0.14 ^d^	1.29 ± 0.08 ^g^
Cooked pasta		
C-F	-	-
C-L	-	-
C-H	-	-
B2.5-F	0.91 ± 0.04 ^g^	2.57 ± 0.18 ^a,b^
B2.5-L	0.58 ± 0.03 ^h^	1.84 ± 0.13 ^e,f^
B2.5-H	-	-
B5-F	5.07 ± 0.25 ^b^	2.72 ± 0.18 ^a^
B5-L	4.12 ± 0.21 ^c^	1.94 ± 0.14 ^d,e^
B5-H	2.93 ± 0.14 ^d^	1.72 ± 0.13 ^e,f^

Lowercase letters indicate a significant difference (Fisher’s LSD test at *p*-value < 0.05) between samples.

**Table 4:** The carotenoid contents of broccoli leaf powder (BLP) and pasta products, expressed as mg/kg DW.

	Violaksantin	Astraksantin	Capsantin	Lutein	Cryptoxanthin Derivative	α-Cryptoxanthin	β-Cryptoxanthin	δ-Carotene	γ-Carotene	ζ-Carotene	β-Carotene
BLP	12.96 ± 0.65	1.23 ± 0.10	29.47 ± 1.77	738.77 ± 36.94	9.26 ± 0.74	13.64 ± 0.82	41.19 ± 2.06	236.52 ± 18.92	92.03 ± 5.52	51.24 ± 2.56	982.09 ± 78.57
Uncooked pasta											
C-F	-	-	-	-	-	-	-	-	-	-	-
C-L	-	-	-	-	-	-	-	-	-	-	-
C-H	-	-	-	-	-	-	-	-	-	-	-
B2.5-F	-	-	-	133.52 ± 10.68 ^b^	0.72 ± 0.04 ^d,e^	0.42 ± 0.02 ^e^	1.09 ± 0.09 ^c^	1.39 ± 0.12 ^d^	14.36 ± 0.72 ^e^	18.50 ± 1.48 ^c^	112.44 ± 6.75 ^b^
B2.5-L	-	-	-	32.30 ± 2.58 ^f^	0.70 ± 0.04 ^d,e^	0.72 ± 0.04 ^d^	0.49 ± 0.04 ^d^	-	25.20 ± 1.26 ^b^	13.66 ± 1.09 ^d^	94.32 ± 5.66 ^c^
B2.5-H	-	-	-	72.34 ± 4.34 ^d^	0.44 ± 0.02 ^g^	0.44 ± 0.04 ^e^	0.36 ± 0.02 ^d,e^	-	16.69 ± 1.34 ^d^	11.44 ± 0.69 ^e,f^	86.27 ± 4.31 ^c,d^
B5-F	0.83 ± 0.05 ^a^	0.22 ± 0.01 ^b^	25.56 ± 1.27 ^a^	102.60 ± 6.16 ^c^	0.85 ± 0.04 ^c^	1.12 ± 0.09 ^b^	2.48 ± 0.15 ^b^	5.36 ± 0.27 ^a^	6.44 ± 0.52 ^g^	3.80 ± 0.23 ^h^	68.36 ± 3.42 ^e^
B5-L	-	0.51 ± 0.03 ^a^	5.46 ± 0.44 ^b^	141.56 ± 11.32 ^b^	0.77 ± 0.05 ^c,d^	1.08 ± 0.05 ^b^	2.72 ± 0.22 ^a^	2.51 ± 0.15 ^c^	4.58 ± 0.23 ^g^	3.50 ± 0.28 ^h^	54.14 ± 3.25 ^f^
B5-H	0.88 ± 0.04 ^a^	-	-	138.90 ± 6.95 ^b^	1.12 ± 0.09 ^b^	1.10 ± 0.07 ^b^	1.20 ± 0.06 ^c^	-	13.96 ± 0.84 ^e^	21.10 ± 1.06 ^b^	130.27 ± 10.42 ^a^
Cooked pasta											
C-F	-	-	-	-	-	-	-	-	-	-	-
C-L	-	-	-	-	-	-	-	-	-	-	-
C-H	-	-	-	-	-	-	-	-	-	-	-
B2.5-F	-	-	-	31.28 ± 1.56 ^f^	0.56 ± 0.04 ^f^	0.49 ± 0.03 ^e^	0.33 ± 0.02 ^d,e^	-	35.53 ± 2.13 ^a^	22.23 ± 1.11 ^a^	130.89 ± 10.47 ^a^
B2.5-L	-	-	-	50.67 ± 2.53 ^e^	0.30 ± 0.02 ^h^	0.20 ± 0.01 ^f^	0.29 ± 0.01 ^e^	-	19.27 ± 1.16 ^c^	13.95 ± 0.70 ^d^	88.70 ± 7.10 ^c^
B2.5-H	-	-	-	-	-	-	-	-	-	3.13 ± 0.19 ^h^	-
B5-F	-	0.57 ± 0.05 ^a^	29.63 ± 1.78 ^a^	158.25 ± 7.91 ^a^	1.29 ± 0.10 ^a^	1.70 ± 0.10 ^a^	2.38 ± 0.12 ^b^	1.39 ± 0.11 ^d^	15.82 ± 0.95 ^d,e^	10.53 ± 0.53 ^f^	113.45 ± 9.08 ^b^
B5-L	-	-	-	113.32 ± 6.80 ^c^	1.22 ± 0.06 ^a^	0.84 ± 0.07 ^c^	0.30 ± 0.02 ^e^	-	14.12 ± 1.13 ^e^	11.95 ± 0.72 ^e^	75.06 ± 3.75 ^d,e^
B5-H	0.35 ± 0.03 ^b^	0.23 ± 0.01 ^b^	-	63.54 ± 5.08 ^d^	0.65 ± 0.04 ^e,f^	0.73 ± 0.04 ^d^	1.17 ± 0.09 ^c^	3.17 ± 0.19 ^b^	10.80 ± 0.54 ^f^	6.38 ± 0.51 ^g^	47.25 ± 2.83 ^f^

Lowercase letters indicate a significant difference (Fisher’s LSD test at *p*-value < 0.05) between samples.

**Table 5:** Antioxidant capacity of broccoli leaf powder (BLP) and pasta products.

	TPC *	TEAC ABTS **	FRAP **
BLP	6.76 ± 0.58	6.95 ± 0.82	18.45 ± 0.78
C-F	0.34 ± 0.08 ^d,e^	0.27 ± 0.04 ^d,e^	0.22 ± 0.02 ^e^
C-L	0.29 ± 0.12 ^d,e^	0.18 ± 0.03 ^e,f^	0.21 ± 0.01 ^e^
C-H	0.21 ± 0.05 ^e^	0.13 ± 0.04 ^f^	0.28 ± 0.02 ^e^
B2.5-F	0.58 ± 0.09 ^b,c^	0.58 ± 0.05 ^b^	1.24 ± 0.04 ^c^
B2.5-L	0.44 ± 0.10 ^c,d^	0.42 ± 0.09 ^c^	1.09 ± 0.06 ^d^
B2.5-H	0.39 ± 0.10 ^d^	0.31 ± 0.06 ^d^	1.10 ± 0.03 ^d^
B5-F	0.87 ± 0.10 ^a^	0.86 ± 0.08 ^a^	2.13 ± 0.06 ^a^
B5-L	0.65 ± 0.08 ^b^	0.76 ± 0.04 ^a^	2.11 ± 0.08 ^a^
B5-H	0.58 ± 0.09 ^b,c^	0.63 ± 0.09 ^b^	1.95 ± 0.06 ^b^

Lowercase letters indicate a significant difference (Fisher LSD test at *p*-value < 0.05) between samples. * expressed as mg GAE/g DW. ** expressed as µmol TE/g DM.

**Table 6:** Inhibitory effects of broccoli leaf powder (BLP) and pasta products against AGE formation (BSA-GLU, BSA-MGO) and ACE activity.

	AGEs (% Inhibition)		ACE (IC_50_)
	BSA-GLU	BSA-MGO	
BLP	92.78 ± 0.31	94.75 ± 0.23	5.11 ± 0.03
C-F	9.09 ± 0.59 ^h^	NA	411.15 ± 4.94 ^c^
C-L	16.41 ± 2.87 ^f^	NA	434.62 ± 1.28 ^a^
C-H	11.14 ± 0.19 ^g^	NA	424.62 ± 1.11 ^b^
B2.5-F	71.87 ± 0.68 ^c^	49.83 ± 0.33 ^d^	225.92 ± 3.39 ^f^
B2.5-L	63.89 ± 0.44 ^d^	34.08 ± 3.86 ^e^	268.90 ± 1.74 ^e^
B2.5-H	54.19 ± 0.59 ^e^	47.92 ± 1.99 ^d^	278.51 ± 1.99 ^d^
B5-F	74.14 ± 0.35 ^b^	61.77 ± 0.69 ^c^	193.22 ± 5.40 ^g^
B5-L	77.22 ± 0.02 ^a^	65.11 ± 0.28 ^b^	227.80 ± 1.80 ^f^
B5-H	77.93 ± 0.11 ^a^	69.24 ± 0.08 ^a^	229.10 ± 3.60 ^f^

Lowercase letters indicate a significant difference (Fisher’s LSD test at *p*-value < 0.05) between samples. NA—Not achieved.

## Data Availability

Data are contained within the article.
